# Shifts in food consumption patterns in the Levant: a systematic review of the last six decades

**DOI:** 10.1186/s12966-025-01741-8

**Published:** 2025-04-24

**Authors:** Hanin Basha, Aisha Shalash, Yasmeen Wahdan, Niveen M. E. Abu-Rmeileh

**Affiliations:** 1 Institute of Community and Public Health, Birzeit University, West Bank, Palestine; 2 Department of Public Health, College of Health Sciences, Qatar University, Doha, Qatar

**Keywords:** The Levant, Food Consumption Patterns, Dietary Shifts, Systematic Review

## Abstract

**Background:**

Food consumption patterns have changed tremendously since the mid-twentieth century, with a rapid global nutritional shift raising concerns, particularly in disadvantaged regions such as the Eastern Mediterranean region (EMR). Given that food intake is very context-specific, this research examines food consumption patterns in Jordan, Lebanon, Palestine, and Syria, representing the contemporary Levant region.

**Methods:**

A systematic review was conducted by searching PubMed, EMBASE, Web of Science, and CINAHL. The eligibility criteria were to include only original peer-reviewed observational studies reporting individual-level food consumption among local Jordanians, Lebanese, Palestinians, and Syrians. Extracted data were synthesized through descriptive statistics and presented in tables and charts. The risk of bias was assessed using the tool developed by Hoy et al. for prevalence studies.

**Results:**

A total of 43 articles that measured and reported food consumption at the individual level for the populations in these countries were included. Findings reveal that in the 1960s, diets in the region were primarily local, seasonal, and plant-based, with moderate to low animal product intake. By the 1990s, a noticeable shift occurred, marked by increased consumption of processed foods, refined carbohydrates, and animal products, with minimal increase in fruit and vegetable intake. Most studies were conducted in Lebanon, limiting the generalizability of findings across the Levant countries.

**Conclusion:**

This review presents an understanding of food consumption changes on the level of food items, food groups, and dietary patterns specific to the Levant. Future studies on food consumption patterns should prioritize national surveys using valid, reliable, and cultural-specific measurement tools and provide detailed, age-disaggregated dietary data. Public health interventions are needed to address the ongoing dietary shift, which is unfolding amid political instability, economic crises, and food insecurity.

**Supplementary Information:**

The online version contains supplementary material available at 10.1186/s12966-025-01741-8.

## Introduction

Food intake is a well-established modifiable determinant of human health and a high-priority public health concern [1, 2]. Research shows that food intake influences our health, as inadequate or excessive consumption of certain food components or overall unhealthy dietary patterns can increase the risk of a range of health issues, including nutrient deficiencies, metabolic disorders, chronic diseases, and others [3]. Additionally, the reason for the public health concern is that individuals’ decisions in choosing foods are complex and dynamic actions. Various interactive, evolving, and multi-level factors shape food choices, including individual preferences, sociocultural norms, environmental conditions, and economic status [4].

The types, amounts, frequencies, and overall combinations of foods consumed by populations have undergone remarkable changes throughout human history [1]. The twentieth century witnessed a rapid global nutritional shift, which was marked by major changes in dietary intake and associated health outcomes [1]. This nutrition transition is characterized by a move from whole foods and plant-based diets toward energy-dense foods, refined carbohydrates, animal-based products, and saturated and trans-fats. In addition, it includes the use of modern food components, such as products with hydrogenated fat, as well as processing methods that, for example, alter nutrient composition and reduce fiber content [1, 5]. The major changes in food choices and intake have been attributed to the rise of industrialized food manufacturing [6]. Processed and convenient food items are rapidly and easily made accessible to individuals due to urbanization, contributing largely to reduced consumption of local and home-made foods [5, 7].

While advanced nutrition-related practices have improved food productivity [7] and helped control pandemics [8], they have also contributed to the increased prevalence of nutrition-related health conditions, particularly non-communicable diseases such as cardiovascular diseases, diabetes, and hypertension [1]. This nutrition transition is occurring globally [9], but particularly concerning in regions disadvantaged with pre-existing challenges, such as the Eastern Mediterranean region (EMR), which faces threats to its local food systems and agricultural diversity [10].

Given that food consumption is very context-specific, this research will zoom into four neighboring EMR countries which are Jordan, Lebanon, Palestine, and Syria, representing the contemporary Levant [11, 12]. The Levant, known as ‘Bilad al-Sham’ in Arabic, refers to an area of cultural and geographical habitation located east of the Mediterranean Sea in the Arab world [11, 12]. These four countries have several commonalities with respect to geographical location, diversity of landscapes [13], and socioeconomic status (based on the World Bank classification) [14]. Trends in health conditions are also similar in these countries [15], with ischemic heart disease as the leading cause of mortality [16]. In addition, the ongoing economic and political instability in Jordan, Lebanon, Palestine, and Syria heavily influence food availability and accessibility [10, 17]. Therefore, in addition to the rise of food manufacturing, these structural factors reduce the capacity to protect and promote the Levant’s dietary heritage, ultimately influencing the nutritional intake of populations. As a result, undernutrition and nutrient deficiencies remain unresolved, while food insecurity coexists with overnutrition and chronic diseases [10, 18].

In the Levant, where food is one major heritage, countries share similar food production and consumption patterns with distinctive culinary practices shaped mainly by environmental factors [19]. However, the cuisines of the Levant currently include highly processed food items due to their availability and widespread appeal [18, 20]. This food consumption shift was gradual yet rapid in the EMR, beginning in the second half of the twentieth century and continuing into the early 2000s [21].

Existing literature reports the nutrition transition in the EMR [10], and a review has explored dietary changes and obesity in Arab countries [22]. However, an in-depth, contextual, and population-based understanding of shifts in food consumption patterns remains limited. Addressing this gap would require zooming into a specific context and exploring, in detail, the extent and nature of dietary changes. Such insights would be essential for informing research priorities and guiding public health interventions.

The main objective of this research is to comprehensively describe food consumption trend shifts over time in the Levant. It will describe what food groups, food items, and dietary patterns were present in these countries and how these have changed over time. This exploration will be based on population studies of all age groups. Secondary objectives include examining the assessed dietary concepts, the assessment methods, and the reporting approach of dietary intake, as well as examining dietary intake differences based on gender and populations’ meal patterns.

## Methodology

Systematic reviews are a robust tool for identifying, summarizing, and synthesizing evidence and addressing knowledge gaps [23]. A systematic review was conducted following the Preferred Reporting Items for Systematic Reviews and Meta-Analyses (PRISMA) guidelines found in Supplementary Material 1 [24]. The protocol was registered on PROSPERO (ID: CRD42024499107, Supplementary Material 2). A preliminary search on Google Scholar, PubMed, and PROSPERO identified no existing reviews or registered protocols on food consumption changes in the Levant countries.

### Research question

The study adopted the Condition, Context, and Population framework by Munn et al. [25], focusing on food consumption of local populations in Jordan, Lebanon, Palestine, and Syria.

### Search strategy

The literature search was done in PubMed, EMBASE, Web of Science, and CINAHL Complete in December 2023. The following terms and proper truncation for each database were used: ((food intake) OR (food consumption) OR (food pattern) OR (dietary intake) OR (dietary pattern) OR (dietary habit) OR (nutrition survey) OR (eating pattern) OR (eating habit)) AND ((Palestine) OR ("Occupied Palestinian Territory") OR (Gaza) OR ("West Bank") OR ("East Jerusalem") OR (Lebanon) OR (Syria) OR (Jordan)). EndNote was used to import references retrieved from the selected databases, where duplicates were automatically detected and removed. Two reviewers screened titles, abstracts, and full texts in Covidence [26], resolving conflicts through discussion or consultation with a third reviewer. The reference lists of included studies were hand-searched for further relevant articles.

### Inclusion and exclusion criteria

This review considered original observational studies that measured and reported food consumption on the individual level of the local Jordanians, Lebanese, Palestinians, and Syrians. Food consumption refers to the total intake of food by individuals, encompassing nutrients, food items and food groups, and overall dietary patterns [27, 28]. Food consumption on the individual level can be assessed through five primary methods: food records, 24-h dietary recall (24-HR), food frequency questionnaires (FFQ), diet history, and a food habit questionnaire. Each method is designed differently and can be applied in different ways that serve the research objective [29]. Food intake data analysis can be done at different levels, either by food items, by food groups, or on the meal pattern level [30]. Regardless of the measurement tool used and the presented dietary outcome, this research is focused on the overall food intake of populations in the Levant but not the nutrient-level intake of individuals. Only population-based peer-reviewed journal articles were included without restriction on the publication date. Table 1 provides the detailed inclusion and exclusion criteria.

**Table 1:** Inclusion & exclusion criteria

Category	Inclusion	Exclusion
Publication Type	Peer-reviewed articles published in peer-reviewed journals	grey literature Abstracts Books Conference Proceedings Pre-prints
Variable of interest	Studies that measure and\or report the overall individual-level food consumption (regardless of the method used to collect data, for example: FFQ, 24-h, food diary)	Studies assessing the population’s dietary intake with a predefined tool Studies using tools to measure food intake on the household level Studies that measure or\and report specific food item intake
Study design	Cross-sectional, case–control, cohort Original research articles	Case reports, case series Experimental \ intervention studies: clinical trials, field studies, community studies Reviews: narrative review, systematic review, scoping review, meta-analysis
Context	Jordan, Lebanon, Palestine, or Syria	Studies were conducted in regions other than Jordan, Lebanon, Palestine, and Syria
Study participants	Local populations in Jordan, Lebanon, Palestine, or Syria Healthy population Without restriction on age	Populations not Jordanians, Lebanese, Palestinians, or Syrians Patient-based populations (for example, populations with chronic diseases, infectious diseases, allergies, intolerances and disorders) Specialist populations: athletes (trained or skilled in exercises, sports, or games requiring physical strength, endurance, agility, or stamina, and competes in organized events) Infants exclusively breast-fed or bottle-fed
Study settings	Population-based studies conduction in a house, or school or any other non-healthcare facility	Studies conducted in healthcare settings: hospitals, clinics and other healthcare facilities

### Data extraction and analysis

Data were extracted using Excel, focusing on study and population characteristics, dietary concept of interest, assessment methodology, measurement tool, validity and reliability of the tools, method of reporting food consumption, dietary variables reported, and the amounts or frequency of consumption. The FAO/WHO (Food and Agriculture Organization/WHO) Global Individual Food Consumption Data Tool [28] was used to guide the extraction of food items and food groups for harmonizing food classification, with minor adjustments. For studies investigating dietary patterns using multivariate statistical methods, all identified dietary patterns were extracted.

In studies that reported food consumption amounts by subgroups, a weighted average was calculated to estimate overall population-level consumption. Studies presenting multiple datasets across different periods were analyzed separately, and national surveys were compiled. Descriptive statistics were used to summarize study characteristics, dietary concepts, and assessment methods. Food consumption patterns were examined through tables, charts, and narrative summaries. Weighted averages were calculated for food groups reported in grams, servings, or percentage of energy contribution. To facilitate cross-study comparisons, frequency categories were merged. Dietary patterns were identified based on factor loadings greater than 0.3.

### Secondary outputs

A comparison between men\boys and women\girls was conducted to illustrate the contribution of each food group to their total dietary intake. This was accomplished by merging some food groups based on how they are presented in included studies, followed by aggregating all data for each gender across all time periods and then presenting the data in a bar chart. Populations’ dietary habits related to meal patterns and eating environments were summarized in a table.

### Risk of bias

The risk of bias was assessed using the validated 10-item tool by Hoy et al. [31]. However, this tool was developed for disease prevalence studies and, therefore, was adapted for the focus of this research. Specifically, three items were aligned to allow evaluating studies on dietary assessment rather than disease prevalence. Item 6 on disease case definition was adapted to refer to the identification of dietary concepts of interest. Item 7 was used to evaluate the reliability and validity of dietary measurement tools as the parameter of interest. Item 9 on the prevalence period was used to evaluate the length of the recall period for dietary intake. One reviewer assessed the risk of bias in the included studies.

## Results

### Domain one: study characteristics and populations characteristics

**Fig. 1:**
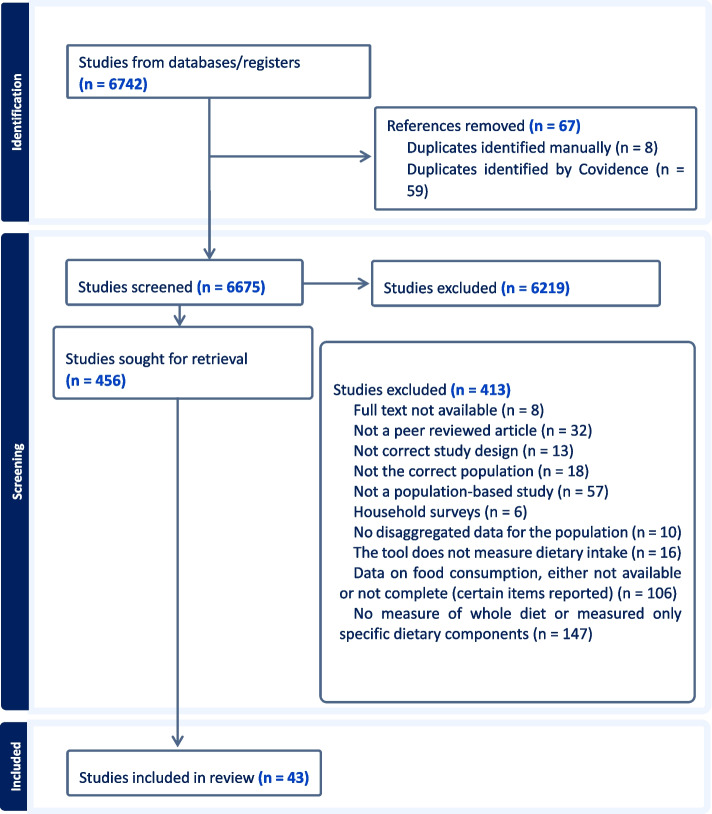
PRISMA Flowchart of included studies

A total of 43 population-based articles that measured and reported food consumption at the individual level were included [32–74]. Supplementary Material 3 is an Excel file with two sheets. The first one is a simple list identifying included studies and the second one is the raw data extraction sheet with the main items extracted from included studies. These articles drew on data from 29 dietary assessments covering 30,009 individuals in the contemporary Levant countries. The PRISMA flowchart outlining the study selection process is shown in Fig. 1. Most excluded studies focused on specific items or nutrients, or dietary data were either partially reported or unavailable.

The included studies were cross-sectional and published between 1964 and 2021, with 30 conducted in Lebanon. In each of Jordan, Palestine, and Syria, three studies were conducted. Three studies included populations from Palestine, Syria, and Jordan, and one study included both Lebanese and Syrians. Table 2 presents a summary of surveys, articles, participants, and the distribution of survey data across life stages, categorized by data collection years. Life stage cutoffs in Table 2 were chosen to simplify data handling and reduce ambiguity. The included studies used varying age group definitions with inconsistent cutoffs, making comparison across populations with the same age group without an overlap extremely difficult. For example, while some studies classified adolescents as individuals aged between 10—19 years, other studies on adults included participants aged 18 years old.

**Table 2:** Summary of surveys, articles, participants, and surveys by age groups across time periods

		**Total**			**Surveys Conducted by Age Group**		
		**Surveys Conducted**	**Total Published**	**# of Participants**	** ≤ 5**	**6–19**	** ≥ 19**
**year data collected**	**1960—1989**	3	3	740	2	3	2
	**1990—1999**	3	5	3,603	1	2	1
	**2000—2009**	4	14	5,429	0	2	1
	**2010—2019**	14	19	10,873	4	3	12
	**2020—2021**	5	6	9,364	1	1	3
**Total**		**29**	**43**	**30,009**	**8**	**11**	**19**

### Domain two: dietary concepts and methodological approaches

Table 3 summarizes assessment methodologies, measurement tools, their validity and reliability, and the reporting approach for dietary data. Face-to-face interviews were the most common methodology, with FFQs and 24-HRs as the primary measurement tools. Validity and reliability were rarely mentioned except in some surveys. The most common reporting approach was the average percentage contribution of food groups to total caloric intake.

**Table 3:** Food intake assessment methodologies, measurement tools, validity and reliability of measurement tools

Categories	Frequency
**Assessment methodology**^a^	
face-to-face interviews	19
self-administered questionnaire	5
self-administered questionnaire: online survey	4
not mentioned	1
**Measurement tool**^a^	
Food frequency questionnaire	11
24-h dietary recall	10
24-h dietary recall and Food frequency questionnaire	2
Dietary habits questionnaire	2
Food consumption survey	2
Eating patterns and food consumption questionnaire	2
**Validity of measurement tools**^a^	
Not mentioned	17
Yes	7
Not validated	6
**Reliability of measurement tools**^a^	
Not mentioned	24
Yes	4
Not validated	2
**Reporting of food consumption**^b^	
Mean daily intake of food groups: percentage of total energy intake (%EI)	16
Factor loading matrix of the main dietary patterns prevalent among study participants	13
Frequency consumption of food groups	6
Mean daily food group consumption: servings	5
Mean daily food group consumption: grams	4
Consumption of food groups: Yes \ No	3
Daily consumption of food groups: adequate \ inadequate	1
**Total of reporting**	**48 **^b^

^a^ These variables were extracted for each data collection, and studies on national surveys were compiled. Thus, the total (29) is equal to the number of times data was collected

^b^ The total exceeds the number of included studies because some studies reported food consumption in two ways

The included studies were not only aimed at exploring and describing food consumption, most of which were concerned with associating dietary intake with various health outcomes (e.g., anthropometrics, diseases). The dietary concepts each study sought to assess, and the corresponding assessment methods generally reflected the specific aims of each study. However, from the included studies, terms were operationalized inconsistently and in multiple ways. Therefore, it was necessary to examine this aspect. Four key concepts emerged from combining equivalent terms: Dietary patterns, Food\dietary consumption\intake, Food\dietary\eating habits, and Food consumption patterns.

Table 4 presents the concepts of interest cross-tabulated with assessment methodology, measurement tool, and reporting approach, describing how each concept was assessed, measured, and reported across each period. National surveys were not handled as a single unit because they utilized different terms and reporting methods, which needed to be reflected in the analysis. When multiple concepts were used within a single study, each was matched with its respective assessment and presentation approach.

- *Food\dietary consumption\intake:* All methodologies and tools were used to assess food intake, with the mean daily percentage contribution of food groups to total energy intake being the most common reported outcome. After 2020, the focus shifted to mean daily servings.
- *Dietary (or feeding) pattern:* Most frequently, dietary patterns were measured using FFQs. Studies in the 1960s interviewed participants about their food patterns and narrated their daily food choices, including detailed explanations of how and when food was consumed. In these studies, the term dietary pattern was used interchangeably with dietary habits. After 2000, dietary patterns were more thoroughly assessed using statistical methods to derive those patterns.
- *Food consumption patterns:* Studies after 2000 assessed food consumption patterns through 24-HR or FFQs. The reported outcome was the mean daily percentage contribution to total energy intake, mean daily servings consumption, frequency of consumption, or factor loading matrices.
- *Food\dietary\eating habits:* Food habits were assessed before 2000 in interviews and narrated. In 2020, using an online survey and a 24-HR, dietary habits were measured, and the reported outcome was the mean consumption of daily servings.

**Table 4:** Cross-tabulation of concepts with methodology and reported outcome

		Dietary Pattern	Food Consumption	Food Habit	Food consumption patterns
**1960–1989 (total # of publications for datasets conducted in this period: 3)**					
**Assessment methodology**	Face-to-face interviews	2	3	2	-
**Dietary Measurement**	Food consumption survey	-	2	-	-
	FFQ	-	1	-	
	Dietary habits	2	-	2	-
**Food groups reporting**	Mean daily intake of food groups (%EI)	-	2	-	-
	Mean daily consumption in servings	-	1	-	
	Narrative description of foods consumed	2	-	2	-
**1990–1999 (total # of publications for datasets conducted in this period: 5)**					
**Assessment methodology**	Face-to-face interviews	-	5	1	-
**Dietary Measurement**	24-HR	-	5	1	-
**Food groups reporting**	Mean daily intake of food groups (%EI)	-	5	-	
	Narrative description of foods consumed	-	-	1	-
**2000–2009 (total # of publications for datasets conducted in this period: 14)**					
**Assessment methodology**	Face-to-face interviews	6	7	-	2
	Self-administered questionnaire	-	1	-	-
**Dietary Measurement**	24-HR	-	6	-	1
	FFQ	6	2	-	1
**Food groups reporting**	Mean daily intake of food groups (%EI)	-	5	-	2
	Dietary patterns	6	3	-	-
	Mean daily consumption in grams	-	-	-	1
	Frequency consumption of food groups	-	1	-	-
**2010–2019 (total # of publications for datasets conducted in this period: 19)**					
**Assessment methodology**	Face-to-face interviews	5	13	-	1
	Self-administered questionnaire	3	4	-	1
	Not mentioned	-	1	-	-
**Dietary Measurement**	24-HR	1	10	-	1
	FFQ	7	6	-	1
	Dietary habits questionnaire	-	2	-	-
**Food groups reporting**	Mean daily intake of food groups (%EI)	-	6	-	1
	Dietary patterns	7	3	-	1
	Mean daily consumption in servings	-	1	-	-
	Consumption of food groups: Yes \ No	-	2	-	-
	Mean daily consumption in grams	-	2	-	-
	Daily consumption of food groups: adequate \ inadequate	-	1	-	-
	Frequency consumption of food groups	-	3	-	1
	Narrative description of frequency of consumption	1	-	-	-
**2020–2021 (total # of publications for datasets conducted in this period: 6)**					
**Assessment methodology**	Face-to-face interviews	-	1	-	-
	Online survey	2	2	1	3
**Dietary Measurement**	24-HR	-	1	-	-
	FFQ	-	-	1	3
	Eating patterns and food consumption questionnaire	2	2	-	-
**Food groups reporting**	Mean daily consumption in servings	2	2	1	1
	Consumption of food groups: Yes \ No	-	1	-	-
	Frequency consumption of food groups	-	-	-	2

### Domain three: food consumption trends

**Fig. 2:**
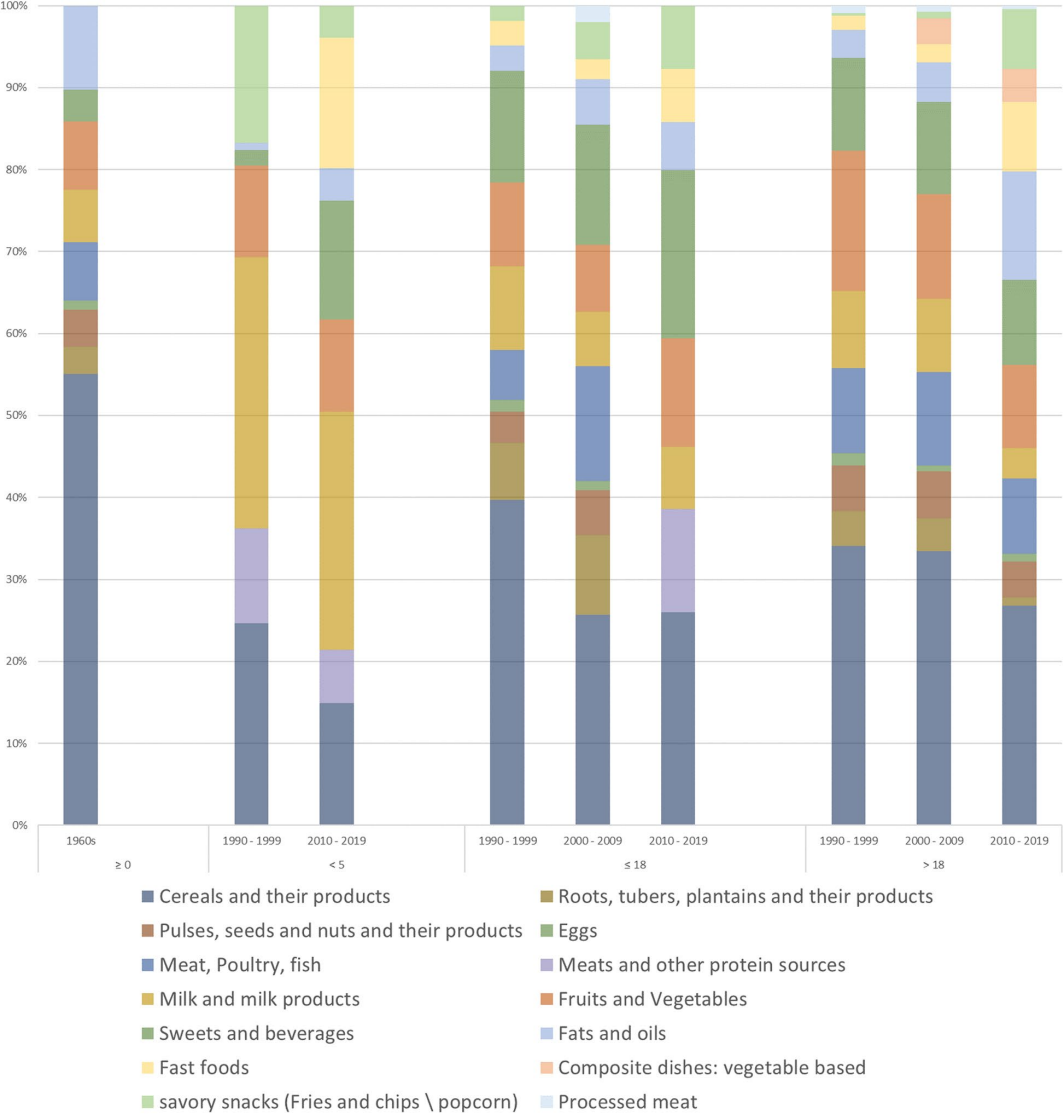
Percentage contribution of each food group to the total energy intake by periods and age groups

Food consumption patterns were examined both narratively and by quantitative analysis. Table 5 presents the food groups and items reported in the included studies across each time period. The ratio represents the proportion of studies reporting on each food group relative to the total number of studies conducted during this period. A higher ratio could identify a higher focus on this food group, while a lower ratio could indicate either a lower interest or an underreporting. Figure 2 is a stacked bar chart that illustrates the percentage contribution of each food group to the total energy intake across all studies that reported this value. Data is presented by decade and divided by age groups.

**Table 5:** Distribution of reported food groups in studies across different periods

**Periods**		**1960—1989**		**1990—1999**		**2000—2009**		**2010—2019**		**2020—2021**
Food Groups	**Ratio**	**Total Studies: 3**	**Ratio**	**Total studies: 3**	**Ratio**	**Total studies: 4**	**Ratio**	**Total studies: 14**	**Ratio**	**Total studies: 5**
Cereals and their products	1.00	. Cereals . High extraction wheat bread	1.00	. Whole grains . Refined grains (rice, pasta, bulgur, bread)	1.00	. Whole grains . Refined grains (rice, pasta, bulgur, bread, ready-to-eat cereals)	1.00	. Whole grains . Refined grains (rice, pasta, bulgur, bread, ready-to-eat cereals)	1.00	. Whole grains . Refined grains (rice, pasta, bulgur, bread, ready-to-eat cereals)
Roots, tubers, plantains	1.00	Starchy vegetables	1.00	Starchy vegetables	1.00	Starchy vegetables (potato)	0.50	Starchy vegetables (potato, corn, parsnips, pumpkin, squash)	0.20	
Pulses, seeds and nuts	1.00	. Pulses: lentils, chickpeas (hummus), beans and falafel . Nuts . Seeds	1.00	. Legumes . Nuts . Seeds	1.00	. Legumes (lentils, hommos, falafel and fava beans) . Nuts . Seeds	1.00	. Legumes (hummus, falafel, broad beans, peas) . Nuts . Seeds (roasted unsalted and raw nuts and seeds)	0.80	. Legumes (beans, lentils, chickpeas) . Nuts
Meat, Poultry, Fish, Eggs	1.00	. Meat (Specially mutton) . Poultry . Fish . Eggs	1.00	. Meat and meat organs . Poultry and poultry organs . Fish . Eggs	1.00	. Meat (beef mostly consumed compared to lamb) . Poultry . Fish . Eggs	1.00	. Meat (specially beef) and organ meats . Poultry . Fish . Eggs	1.00	. Meat (beef, veal, pork, lamb, mutton, horse, and goat) . Poultry . Fish . Eggs
Milk and milk products	1.00	. Milk . Dairy products (powdered milk, labneh, yogurt, cheese) . Breast Milk . Infant formula	1.00	. Milk . Dairy products (powdered milk, labneh, yogurt, cheese) . Sweetened products (frozen yogurt, fruit yogurt, puddings) . Breast Milk . Infant formula	1.00	High and low-fat dairy: . Milk . Dairy products (powdered milk, labneh, yogurt, yellow and white cheese) . Sweetened products (frozen yogurt, fruit yogurt, puddings, milkshake) . Breast Milk . Infant formula	0.86	High and low-fat dairy: . Milk . Dairy products (powdered milk, labneh, yogurt, cheese) . Sweetened products (sweetened milk, whipped cream) . Breast Milk . Infant formula	1.00	. Milk . Dairy products (yogurt, cheese, creamy cheese)
Vegetables	1.00	Fresh, dried, pickled vegetables (tomato, cucumber, turnip, carrots, green, leafy wild plants)	1.00	Vegetables and their products	1.00	Vegetables and their products	1.00	Vegetables and their products	1.00	Vegetables and their products
Fruits	1.00	Seasonal fruits (citrus fruits)	1.00	. Whole fruits . Fresh fruits . Dried fruits	1.00	. Whole fruits . Fresh fruits . Dried fruits	1.00	. Whole fruits . Fresh fruits . Dried fruits	1.00	Fruits and their products
Sweets and Beverages	0.67	. Added sugars and jams . Hot Beverages (Coffee, Tea)	1.00	. Added sugars, jams, honey, molasses . Traditional sweets . Sweets (pastries, candies, biscuits, cakes, ice cream) . Sweetened juices, regular soft drinks . Hot Beverages (Coffee, Tea) . Alcoholic Beverages	1.00	. Added sugars, jams, honey, molasses . Traditional sweets (kunafeh) . Sweets (pastries, candies, biscuits, cakes, ice cream, chocolates, jelly, cookies) . Sweetened juices, regular soft drinks . Carbonated beverages . Hot Beverages (Coffee, Tea) . Alcoholic Beverages	0.79	. Added sugars, jams, honey, molasses . Traditional sweets . Sweets (pastries, candies, biscuits, cakes, ice cream, chocolates, jelly, cookies, donuts, muffin, croissant, popsicle, cereal and nutrition bars) . Sweetened juices (bottled fruit juices, canned fruit juices, lemonade, fruits canned in heavy syrup) . Carbonated beverages . Energy drinks . Unsweetened beverages (diet sodas and any beverages made with non-nutritive sweeteners . Hot beverages (coffee, tea, Nescafe, hot chocolate) . Alcoholic beverages	0.20	. Traditional sweets . Sweets (ice cream, cake, chocolate) . Canned juice . Carbonated beverages . Hot beverages (coffee, tea)
Condiment	0.00		0.33	. Mayonnaise . Salad dressings	0.25	. Mayonnaise . Salad dressings	0.43	Condiments (mayonnaise, ketchup, mustard) . Chicken/meat stock . Ground thyme	0.00	
Fats and oils	1.00	. Fried mutton . Unsaturated fats (olive oil, tahini))	1.00	Fats and oils (Olive oil, olives, avocado, sesame butter, all kinds of oils, animal-based fat)	0.75	Fats and oils (butter, sesame paste, olive oil and ghee)	0.79	. Saturated oils: butter . Vegetables oil: olive, corn, sunflower, soy . Ghee . Tahini . Peanut butter	0.20	Fats
Traditional composite dishes	1.00	Traditional Middle Eastern foods . Majaddarah (lentils and rice) . Kishk (yogurt and wheat) . Hummos (chickpeas and tahini paste)	0.33	. Rice-based dishes . Legume-based dishes . Potato-based dishes . Vegetable-based traditional dishes . Dairy products and yoghurt-based dishes	0.50	. Potato-based dishes . Vegetable-based dishes . Macaroni and cheese	0.29	. Vegetables and vegetable-based dished . Lebanese mixed dishes . Meat and poultry-based traditional dishes . Legume-based traditional dishes . Rice and rice-based dishes . Pasta-based dishes . Macaroni and cheese . Spaghetti lasagna	0.00	
Fast food	0.00		0.33	Fast food (ready to eat sandwiches, burgers, pizzas, pies, pastries)	0.50	fast foods Fast food (ready to eat sandwiches, burgers, pizzas, pies, pastries, shawarma, falafel sandwiches)	0.64	fast foods Fast food (ready to eat sandwiches, burgers, pizzas, pies, pastries, shawarma, falafel sandwiches)	0.20	Pastries and pizza
Savory snacks	0.00		1.00	. Chips and salty crackers (Chips, pretzels, popcorn)	0.50	. Chips and salty crackers (Chips, pretzels, popcorn) . Fried potato	0.71	. High fat, high salty snacks (crackers, potato chips, pretzels, popcorn) . Fried potatoes	0.20	Chips and salty crackers (Chips, pretzels, popcorn) . Fried potato
Processed meats	0.00		0.33	Processed meat	0.50	Processed and canned meat	0.36	. Processed meats (luncheon meats, hotdog, sausages) . Canned meats	0.20	Processed meats
Miscellan-eous	0.67		0.33	Pickles, soups, broth	0.25	Pickles, soups, broth	0.07	Pickled turnips	0.00

### Food consumption trends shifts

The earliest studies from the 1960s and 1980s, and among some populations in the 1990s, showed that diets were primarily reliant on homemade, local, and seasonal food availability. Populations primarily derived their energy intake from cereals, while their protein sources were whole grains and legumes, with weekly intake of meats, primarily mutton. Milk and milk products that were basically yogurt, labneh, and cheese, in addition to fats and oils, were also staples in the diet. Fruit and vegetable consumption depended on seasonal availability, with certain locations having an abundance during specific seasons while others experienced scarcity. Sugar intake was limited to table sugar used in Turkish coffee and jams.

These food groups reported in earlier studies remained constant, but the shift in trend was observed starting from the 1990s with variations across age groups. However, there are generally similar changes. Initially, the overall trend showed an increased diversity in assessed and reported food groups and items and an expansion in food choices beyond the local items, except for the latest studies where the assessment and reporting of food groups and items was limited. The dominance of cereals in the diet and reporting of starchy vegetables and oils decreased over time. Fruits, vegetables, legumes, and dairy contributed more to total energy intake, but their frequency of consumption across different studies showed a decrease. Sweets and sweetened beverages, fast foods, savory snacks, and processed meats food groups emerged and started to constitute more of the populations’ food intake (in descending order). Studies in 2020 and 2021 have minimal and varied outcomes, but generally, the observed influence of the pandemic appears to be an overall increase in food consumption.

### Consumption and characteristics of traditional dietary patterns

The earliest studies characterized the local dietary patterns, and after 2000, several studies identified dietary patterns among populations, often labeling them as traditional based on their components. While the traditional dietary pattern, characterized by the same previously mentioned items, was present in some study populations, it was not present among all populations. Additionally, some identified patterns included a mix of traditional food items and other processed options. This suggests that while the traditional dietary pattern is present to some extent, it has changed over time.

### Food-insecure households

Five studies conducted after 2014 examined food insecurity in Palestine and Lebanon [36, 40, 41, 51, 74]. These studies highlight the concern of household food insecurity among children, adolescents, and mothers. In summary, participants from food-insecure households had lower total energy intake, lower adherence to the Mediterranean dietary pattern, higher risk of suffering from dietary inadequacy, and a higher prevalence of malnutrition.

### Secondary outcomes

Eating behaviors were examined to provide a comprehensive understanding of food consumption patterns (details in Supplementary Material 4). A shift in meal patterns was observed. Early studies consistently reported three daily homemade meals with snacks, a trend that persisted over time. However, post- 2000 studies noted increased meal skipping, particularly among adolescents and adults. Weekly reliance on restaurant foods also became more common across all age groups.

Gender differences in food consumption were analyzed using studies reporting percentage energy contributions (Supplementary Material 5). Women\girls derived more energy from fruits, vegetables, dairy, sweets, and sweetened beverages, favoring plant-based diets. Men\boys consumed more energy from cereals, animal products, and ready-to-eat meals, favoring Westernized diets.

### Quality assessment of included studies

The risk of bias is presented in Supplementary Material 6. The primary concern in studies was the validity and reliability of dietary measurement tools. A few national surveys used valid and reliable dietary measurement tools. Other studies either did not mention anything about the validity of the tools or said the tool was not validated. Some of which reported the use of approaches to enhance data accuracy, including conducting a pilot study, training field workers, using tools to visualize food amounts, and following the multiple-pass approach by the United States Department of Agriculture (USDA). Non-response bias was common due to the lack of reporting response rates. Studies conducted during COVID- 19 had a moderate risk of bias, primarily due to the online assessment method with convenience sampling, which introduced selection bias. Additionally, participants had to recall dietary intake before COVID- 19, increasing the likelihood of recall bias.

## Discussion

### A brief summary

Forty-three studies were included in this review, with a total of 30,009 individuals in the Levant. Data collection spanned from the early 1960s till 2021, most of which were in Lebanon. Face-to-face interviews were the most common methodology for data collection, with FFQs and 24-HRs as the primary measurement tools. The most common reporting approach was the average percentage contribution of food groups to total caloric intake. Findings reveal that in the 1960s, diets in the region were primarily local, seasonal, and plant-based, with moderate to low animal product intake. By the 1990s, a noticeable shift occurred, marked by increased consumption of processed foods, refined carbohydrates, and animal products, with minimal increase in fruit and vegetable intake.

### Food consumption trends

Food shortage and malnutrition were widespread globally until the 1960s, a period marked by severe famines in many regions [5, 8]. Around this time, Popkin identified the receding famine phase, during which improved food availability began to emerge [1]. By the 1960s, some Middle Eastern regions had already overcome calorie deficits and undernourishment [75]. In developing countries, including the Middle East, cereal and grain production, importation, and consumption increased significantly from the 1950s to the 1980s, with wheat being a dominant staple [76]. Food aid, economic development, and increased demographic density were significant short-term driving factors for this increase [76].

The dietary pattern identified among populations in the Mediterranean region shares similarities with the traditional pattern identified in the Levant in this systematic review [77]. A review article by Amr [78] offers a close look at the local traditional foods in the Levant region before the year 2000 [78]. Through site observations and existing literature, the review provides an understanding of the seasonal production of fruits, vegetables, cereals, and milk and how local populations adopted techniques such as drying, pickling, and fermentation to preserve food and prepare ready-to-eat items during abundance for availability during scarcity. It should be noted that although alcohol consumption has been reported across some of the included studies, especially after the year 2000, it could be under-reported. In Arab countries, it is generally considered to be a social taboo, even if it is not prohibited [79].

Findings from previous studies corroborate our findings, with similar patterns documented in other Mediterranean countries [80]. For example, populations in Greece are transitioning towards the Westernized diet [81], with decreased consumption of whole-grain bread, fruits, vegetables, and legumes and increased meat intake from 1960 to 2005 [77]. Similarly, in Spain, adherence to the Mediterranean diet began to decline around 1999, particularly among younger generations [82]. Therefore, shifts in food consumption patterns have been widely documented, marked by a move away from local, minimally processed foods to energy-dense, industrially processed items with lower nutritional value [83]. This dietary transition had far-reaching implications for populations’ health. For example, ultra-processed foods, often containing chemical additives to enhance preservation, texture, and flavor, increase the risk of cardiometabolic and common mental disorders based on an umbrella review of meta-analyses [84].

This dietary transition has been facilitated in low- and middle-income countries by urbanization and the modernization of food systems driven by industrialization [83]. However, the transition in the Levant has been compounded by political instability, economic crises, and food insecurity. These factors exacerbate pre-existing health conditions in countries still struggling with undernutrition and food insecurity. Therefore, dietary shifts should be addressed with political commitment and community involvement [9].

### General characteristics of included studies

There has been a global increase in expenditure on research and development in the twenty-first century, contributing to the growth of scientific publications, with a pronounced expansion after 2010, particularly in middle-income countries [85, 86]. Nutrition research has evolved and shifted from nutrient-level assessments, particularly vitamins, in the early twentieth century [87] towards dietary patterns by the late twentieth century [88]. This could explain the absence of studies conducted before the 1960s in this systematic review and the increase in the number of studies over time. Research in low- and middle-income countries can be challenging due to many barriers like limited resources and poor national infrastructure and investment in research [89]. In Arab countries, research is further hindered due to armed conflicts [90, 91] as well as poor dissemination of research findings and weak institutional collaboration [92]. The notable number of publications from Lebanon can be attributed to the strong academic institutions in public health and nutrition research, like the American University of Beirut [93].

The analysis of epidemiological patterns is generally hindered due to the unavailable standard age classification [94], which was apparent in this review. Given that dietary needs vary greatly across different life stages, breaking down health data is essential in research, and this breakdown should be harmonized to allow comparability. Diaz et al. [94] proposed an age grouping of 5-year intervals with more detailed categories for children under five since their development is rapid [94]. These aspects highlight the need to support dietary research, particularly in Jordan, Palestine, and Syria, where research is very limited compared to Lebanon. Research barriers should be addressed with increased investment in funding and institutional collaboration. Additionally, adopting standardized age classification in presenting dietary data is essential to allow comparability at the national, regional, and international levels.

### Assessment methods and dietary concepts

Dietary intake assessment methods depend on research aims and design [29, 95]. Face-to-face interviews are the gold standard classical approach, compared to online assessment, which increases the risk of selection bias, limiting generalizability [96]. However, web-based dietary intake data collection could be promising in providing good data in epidemiological studies [97] and increasing feasibility for participants [96]. There are several methods for measuring individual dietary intake data [30], most of which were used in included studies. Employing two dietary measurement methods is recommended to enhance data accuracy [98]. However, the most accurate method is an actual weighing of food to be consumed [99], which was employed only in the earliest included studies in the 1960s. The choice of method to assess and present dietary intake data impacts the understanding and implications. While the variation across the included studies limited the ability to conduct a comprehensive quantitative analysis, it provided a wider understanding of dietary intake in terms of quantities and frequencies. Faber et al. [100] highlighted the different implications of each method to present dietary data and how the choice should be based on the study objectives, arguing that in research articles sufficient information should be provided to ensure clarity and enable comparison across studies [100].

A major limitation is that most studies either did not report information about the validity and reliability of measurement tools or stated the tools were not validated. The widely recognized USDA 5-step multiple-pass method for 24-HR, while effective in some contexts [101, 102], has not been consistently validated across diverse populations, especially those consuming mixed dishes [103]. Ensuring valid and reliable tools is essential for accurate dietary intake measurements [104]. Recently, several measurement tools in the Levant have been developed and validated [105–111], including a photographic food atlas for the EMR [112]. In summary, using valid, reliable, and contextual measurement tools while presenting sufficient dietary information should be prioritized in dietary research. If feasible, face-to-face interviews with the actual weighing of food or employing two assessment methods are also supported approaches. These can be promising in improving data accuracy and allowing comparability.

Conceptually, dietary patterns refer to the ability to capture the totality of food consumption, but in terms of assessment and presentation methods, it has evolved. A study conducted in the 1960s assessed and reported dietary patterns for men of Japanese ancestry [113], using dietary recalls and consumption frequency of food items. This research categorizes food items based on previous knowledge of Japanese and Western dietary components, and dietary data was presented in terms of average consumption in grams and percentage of frequent consumers of each item in each identified dietary pattern. Another research conducted in the 1970s used both qualitative and quantitative approaches to present food groups and items consumption of populations [114]. The shift towards capturing the complexity of total food intake using multivariate statistical methods began in the 1980s [27, 115].

Dietary habits, often used interchangeably with dietary patterns, refer to activities related to dietary intake, including food group consumption, food safety practices, and eating regular meals [116]. Dietary habits questionnaires might be used to collect data related to food consumption [30]. The included studies used this concept to assess populations’ dietary intake, describing food groups'consumption habits, frequencies, or amounts in servings, in addition to other practices. Included studies assessed food consumption patterns most commonly using FFQs and presented dietary data using a variety of approaches. Food consumption patterns reflect populations’ eating trends in relation to the food system and how changes occur in response to changes in the economic and agricultural sectors [117].

### Implications from this review

The Levant countries have structural factors, including unstable political and economic conditions, scarce resources, and ongoing conflicts, which are barriers to accessing and consuming healthy food options, leading to heightened food insecurity [118, 119]. These structural factors are persistent concerns in these countries. In Gaza, vegetables were once sold at extremely low prices in early 2000. However, the devastating humanitarian crises, further exacerbated by the last war, have caused widespread severe destruction to the food system and starvation [120]. Similarly, Syrians have suffered severe conflict-driven food insecurity and hunger [121]. In addition to these conflicts, unilateral coercive measures, such as economic sanctions, have further impacted food security, especially in Syria, by restricting access to essential goods. This worsened economic instability and deepened the vulnerability of disadvantaged populations [122].

Amidst the structural factors and ongoing globalization, several aspects need to be addressed in the Levant. Foremost, the food crisis among the most disadvantaged populations can only be addressed when tackling the longstanding military violence [119, 121, 123]. Additionally, the United Nations Human Rights Council's decision on not to use unilateral coercive actions that affect food security, should be respected [122]. Moreover, subsidizing healthy food items in supermarket settings or using targeted voucher programs could play a key role in improving dietary intake, as demonstrated in field experiments [124] and a systematic review and meta-analysis [125]. These two articles suggest that price reduction on healthier foods, particularly fruits and vegetables, can significantly increase their purchase and consumption.

Further comprehensive dietary assessments in the Levant countries are crucial for a better understanding of current patterns and for informing long-term effective cultural interventions for enhancing dietary intake. Additionally, such assessments can identify vulnerable populations, as structural factors disproportionately influence certain populations [126]. For instance, in these countries, Palestinian refugees, the people of Gaza, and residents of Area C in the West Bank, Palestine, are among the most vulnerable populations, facing severe political and economic hardships, with high humanitarian needs [127].

### Research strengths and limitations

This systematic review provides a detailed and contextual understanding of food consumption changes in the Levant countries. It builds on population-based research conducted among locals in this region. This review addresses gaps in knowledge, pointing out what needs to be prioritized and accomplished in future research on food consumption. However, there are several limitations. Initially, this review relied on published data without requesting raw datasets from authors. Only peer-reviewed studies that measured and reported individual-level food consumption were included, excluding household-level studies, grey literature, and studies focusing solely on specific food items or groups. Consequently, the review may lack comprehensive coverage.

Other limitations are related to included studies, as only a few national surveys employed valid and reliable dietary measurement tools, and the variation in these tools leads to inconsistent dietary data presentation and the potential underreporting of certain food groups and items. Most studies were conducted among Lebanese populations, with fewer studies from other Levant countries. The overrepresentation of Lebanon in this review would affect the generalizability of the findings. The analysis by age subgroups was also limited due to overlapping age categories. Moreover, given that there was a very limited number of studies conducted in the twentieth century, our understanding of food consumption patterns before the nutrition transition might not be representative and inclusive.

## Conclusion

This systematic review offers an understanding of how food consumption trends have shifted in the Levant countries. It builds on 43 population-based cross-sectional studies that measure and report the overall food consumption among Jordanian, Lebanese, Palestinian, and Syrian populations. While the focus is on food consumption trends, the review examined dietary concepts, assessment methods, and approaches to present dietary data. In the 1960s, populations relied on local, seasonally available, and homemade foods, with high reliance on plant-based items and moderate to low consumption of animal products. By the 1990s, dietary patterns started to shift towards increased reliance on commercially processed and industrialized foods, refined carbohydrates, and animal products, accompanied by a modest but not sufficient rise in fruit and vegetable consumption. This systematic review also highlights some gaps in dietary assessment research in the region, addressing the need for national and representative research, which prioritizes assessing food consumption using valid, reliable, and context-specific measurement tools with comprehensive, detailed, and age-disaggregated presentation of dietary data. Furthermore, in these countries, tackling the longstanding military violence, not using unilateral coercive actions, and subsidizing healthy food items are essential actions that should be addressed and supported by a political well to alleviate food insecurity and enhance the dietary patterns of populations.

## Supplementary Information

Supplementary Material 1.

Supplementary Material 2.

Supplementary Material 3.

Supplementary Material 4.

Supplementary Material 5.

Supplementary Material 6.

## Data Availability

No datasets were generated or analysed during the current study.

## Abbreviations

24-HR: 24 Hour Recall
CIHEAM: International Centre for Advanced Mediterranean Agronomic Studies
EMR: Eastern Mediterranean Region
FAO: Food and Agriculture Organization
FFQ: Food Frequency Questionnaire
PRISMA: Preferred Reporting Items for Systematic reviews and Meta-Analyses
USDA: United States Department of Agriculture
WHO: World Health Organization
