# Supplementary material for: Shifts in food consumption patterns in the Levant: a systematic review of the last six decades
Source: Int J Behav Nutr Phys Act. 2025 Apr 24;22:50. doi: 10.1186/s12966-025-01741-8 (PMC12023382; doi:10.1186/s12966-025-01741-8)
Supplement: Supplementary file 2 — Supplementary Material 2. [file 12966_2025_1741_MOESM2_ESM.docx]

# Supplementary Material 2: Systematic Review Protocol

**Dietary Patterns Shifts in Palestine, Lebanon, Syria and Jordan: A Systematic Review**

*Hanin Basha, Aisha Shalash, Yasmeen Wahdan, Niveen Abu-Rmeileh*

**Review question**

How did dietary patterns of local Palestinian, Lebanese, Syrian and Jordanian populations change over time?

**Searches**

To obtain peer reviewed journal articles, the search was conducted using the following electronic databases: PubMed, EMBASE, Web of Science and CINAHL complete.

The following terms were used: ((food intake) OR (food consumption) OR (food pattern) OR (dietary intake) OR (dietary pattern) OR (dietary habit) OR (nutrition survey) OR (eating pattern) OR (eating habit)) AND ((Palestine) OR ("Occupied Palestinian Territory") OR (Gaza) OR ("West Bank") OR ("East Jerusalem") OR (Lebanon) OR (Syria) OR (Jordan)).

**Types of study to be included**

This review will consider only peer reviewed journal articles: population-based observational studies, which include data on dietary patterns of the local Palestinians, Lebanese, Syrians and Jordanians.

Exclusion criteria:

- Abstracts, books, conference proceedings.

- Case reports, case series, experimental\intervention studies, narrative reviews, scoping reviews, systematic reviews and meta-analysis.

- Grey literature.
- Participants who are not the local population in Palestine, Lebanon, Syria or Jordan.

- Patient-based populations (for example: populations with chronic diseases, infectious diseases, allergies, intolerances and disorders).

- Infants exclusively breastfed or bottle-fed.

- Specialist populations (for example athletes).

- Studies conducted in healthcare settings: hospitals, clinics and other healthcare facilities.

- Studies that do not measure and report the overall dietary pattern.

**Condition or domain being studied**

Dietary patterns and the shifts over time will be studied.

**Participants/population**

Local populations in Palestine, Lebanon, Syria and Jordan. No restriction on age.

**Intervention(s), exposure(s)**

The term dietary pattern refers to the overall combination and quantities of foods consumed by individuals. This review will include studies reporting on the dietary patterns of the local Palestinian, Lebanese, Syrian and Jordanian populations.

**Comparator(s)/control**

Not applicable

**Context**

The participants of the studies which will be included should be the local population of Palestine, Lebanon, Syria or Jordan.

**Main outcome(s)**

The shift or change in dietary patterns of the local populations over time.

**Additional outcome(s)**

- Dietary pattern assessment tools.

- Other dietary habits including: food preparation methods and timing of meal consumption.

- Differences across countries.

- Exploration of dietary pattern terminologies.

- Nutrition related health conditions.

**Data extraction (selection and coding)**

- Screening of title and abstracts, and full texts, will be conducted by two independent reviewers. Conflicts will be resolved through discussion between the two reviewers. When an agreement is not reached, a third reviewer will be consulted.

- If information is insufficient in the abstract, full-text will be screened.

- Hand search of references will be done for included articles.

- Data to be extracted: Journal name, authors, data collection year, publication year, country of study, study design, sample size, demographics and participant characteristics (available information for example: age, gender, health status), dietary assessment tool, reliability of the dietary assessment tool, dietary variables (food items, food subgroups and groups, quantities and frequency of consumption, terminology of a dietary pattern as recorded by the researcher, food preparation methods, characteristics of food consumption), and statistical analysis methodology

- Data will be recorded using Excel.

- Data extraction will be conducted by one reviewer for all articles. Additionally, 5% of the articles will be independently extracted by two reviewers to ensure consistency and accuracy of the extraction process.

**Risk of bias (quality) assessment**

The risk of bias for the quantitative observational studies will be assessed using the tool developed by Hoy et al. (2012).

The Critical Appraisal Skills Programme (CASP) Checklist for qualitative research will be used.

**Strategy for data synthesis**

· PRISMA flow chart to indicate the number of studies at each stage.

· For quantitative data, the following variables will be expressed in descriptive tables:

- Study characteristics: year study conducted, country, and the population characteristics as available.

- Study methodology: design, data collection method.

- Food items, food subgroups and groups.

- Frequency of the consumption will be determined per day if possibly, or per week or month.

- Food preparation methods.

- If meals, snacks or other specific food products were part of the data collected, they will be recorded in the table as food items.

· The change in dietary pattern over time will be explored and discussed.

· Comparison of results by countries will be discussed.

· Methods of data collection will be discussed.

· For narrative synthesis, food items, preparation methods, timing of meals and other dietary habits will be extracted as themes, and the content and other details will be coded using MAXQDA software.

**Analysis of subgroups or subsets**

When possible, the dietary pattern variable will be examined by the following subgroups:

· by participants age groups as provided by the studies which will be included, but generally: children, adolescents, adults and elderlies.

· by gender: male and female.

· by terminology of diet: as provided by the studies which will be included.

· by diet related health conditions.
