# Supplementary material for: Shifts in food consumption patterns in the Levant: a systematic review of the last six decades
Source: Int J Behav Nutr Phys Act. 2025 Apr 24;22:50. doi: 10.1186/s12966-025-01741-8 (PMC12023382; doi:10.1186/s12966-025-01741-8)
Supplement: Supplementary file 4 — Supplementary Material 4. [file 12966_2025_1741_MOESM4_ESM.docx]

# Supplementary Material 4: Meal Patterns – Secondary Outcomes

| **First Author** | **Year data collected** | **Sample size** | **Age group** | **Description** |
| --- | --- | --- | --- | --- |
| **Meal Patterns (Frequency of meals and snacks throughout the day)** | | | | |
| Cowan, J. W. | 1962 | 164 | ≥ 0 | Three main meals |
| Cowan, J. W. | 1962-1964 | 167 | ≥ 0 | Three main meals |
| Abudayya, A. | 2002 | 944 | 12 - 15 | Most often skipped meals: breakfast and dinner  38.96% consume 3 meals per day |
| Jaalouk, D. | 2015 | 457 | 18 - 33 | 1 meal 7 (1.5)  2 meals 114 (25)  3 meals 223 (48.9)  4 meals or more 112 (24.6) |
| Al-Awwad, N. | 2015-2016 | 540 | 18 - 25 | One meal 65 (12.1%)  Two meals 256 (47.8%)  Three meals 190 (35.4%)  More than 3 meals 25 (4.7%) |
| **Breakfast consumption** | | | | |
| Abudayya, A. | 2002 | 944 | 12 - 15 | 62.06% consumed breakfast 7 times per week |
| Matta, J. | 2008-2009 | 196 | adults | Breakfast frequency per week (average): 5.1 |
| Jomaa, L. | 2008-2009 | 525 | > 50 | Breakfast frequency per week (average): 5.76 |
| Nasreddine, L. | 2008-2009 | 868 | 6 - 19 | Breakfast consumption per week:  Never: 5.4%  sometimes: 17.9%  Daily: 76.7% |
| Nasreddine, L. | 2011-2012 | 525 | 2 - 5 | Eating breakfast: 6.7536 times per week |
| Musaiger, A. | 2013 | 406 | ≥ 18 | Eating breakfast regularly: 70% |
| **Eating Environment / food source** | | | | |
| Nasreddine, L. | 2008-2009 | 868 | 6 - 19 | Frequency of eating outside home per week:  ≤1 time 47.5%  >1 time 52.5% |
| Nasreddine, L. | 2011-2012 | 525 | 2 - 5 | Eating out: 1.67 times per week |
| Musaiger, A. | 2013 | 406 | ≥ 18 | Bringing food from home: 27.58%  Eating from university’ s cafeteria 86.45% |
