# Supplementary figures and images for: Shifts in food consumption patterns in the Levant: a systematic review of the last six decades
Source: Int J Behav Nutr Phys Act. 2025 Apr 24;22:50. doi: 10.1186/s12966-025-01741-8 (PMC12023382; doi:10.1186/s12966-025-01741-8)

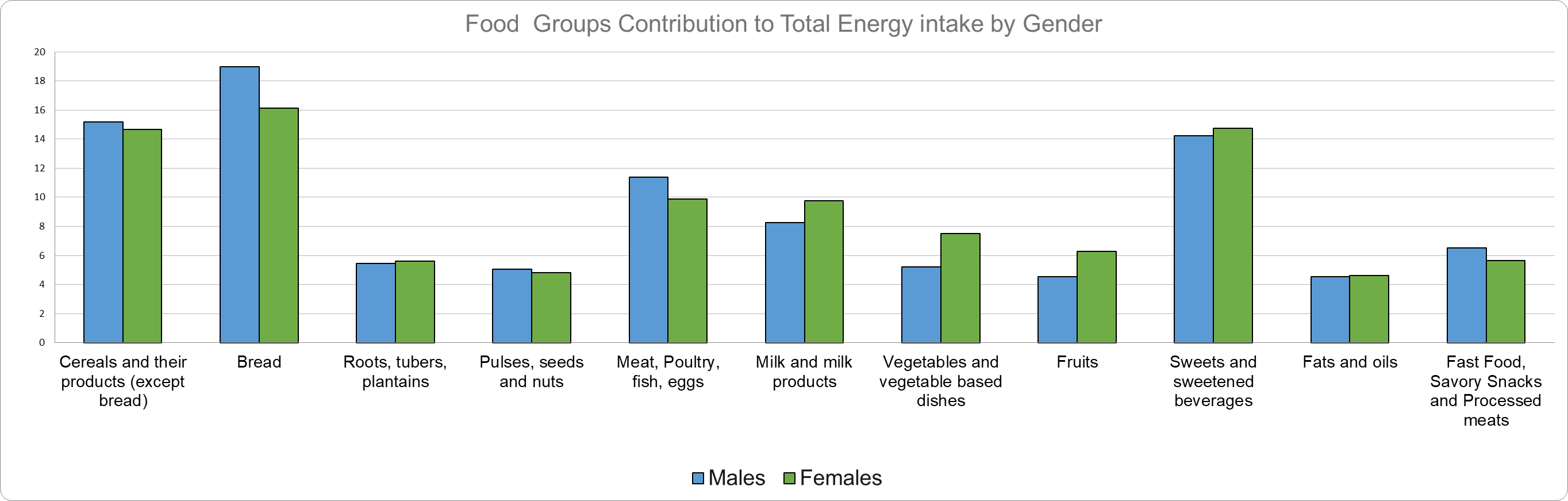

Supplement: Supplementary file 5 — Supplementary Material 5. [file 12966_2025_1741_MOESM5_ESM.png]
